# The Effect of 6‐Week Advanced Oral Care on Oral Microbiome and Mycobiome Composition in People With Dementia Living in Residential Aged Care

**DOI:** 10.1002/cre2.70212

**Published:** 2025-09-08

**Authors:** Sangeeta Khadka, John P. Bowman, Sanjay Gautam, Lynette R. Goldberg, Anna King, Leonard Crocombe, Silvana S. Bettiol

**Affiliations:** ^1^ Centre for Rural Health, College of Health and Medicine University of Tasmania Hobart Tasmania Australia; ^2^ Food Safety and Innovation Centre, Tasmanian Institute of Agriculture University of Tasmania Hobart Tasmania Australia; ^3^ The Peter Doherty Institute for Infection and Immunity Melbourne Victoria Australia; ^4^ Wicking Dementia Research and Education Centre, College of Health and Medicine University of Tasmania Hobart Tasmania Australia; ^5^ Rural Health School, Violet Vines Centre for Rural Research La Trobe University Flora Hill, Bendigo Victoria Australia; ^6^ Tasmanian School of Medicine, College of Health and Medicine University of Tasmania Hobart Tasmania Australia

**Keywords:** bacteria, dementia, fungi, oral cavity, oral hygiene, residential care

## Abstract

**Objectives:**

Oral health is an important aspect of quality of life for older people, especially those with dementia. The impact of an active oral hygiene program on the oral microbiome was explored in a group of older participants (average age 84 years old) with dementia against a separate control group whose oral hygiene followed the status quo.

**Materials and Methods:**

The oral cavity bacteriomes and mycobiomes were assessed from swabs of cheek, gum, and tongue surfaces. Samples were collected at the beginning and end of a 6‐week study period, and bacterial and fungal community profiles were determined by short‐read metabarcode sequencing of 16S and 18S ribosomal RNA (rRNA) genes, respectively.

**Results:**

The predominant bacteria were found to be in order of abundance: *Streptococcus*, *Cellulosimicrobium*, *Rothia*, *Veillonella*, *Prevotella*, *Actinomyces*, and species that belonged to the families Lactobacillaceae and Gemellaceae. Similarly, common fungal species belonged to *Saccharomyces* and the *Candida‐Lodderomyces* clade. The intensive oral hygiene program did not affect bacterial or fungal taxa distributions. A minor reduction in bacterial species richness (15%–20%) was observed post‐intervention in both groups. Mycobiome outcomes varied by sampling sites, unlike bacterial communities, which were relatively homogenous. Participant differences, potentially individual health status, genetics, and other personal factors, explained most data set variations (70%–78% of the variance), with the experimental design accounting for about 2%.

**Conclusions:**

To enable an alteration of oral cavity communities that may improve overall oral health and mitigate infectious disease risks in older people, especially those in residential care, targeted and specific hygiene approaches may be needed for the purposes of assessing effectiveness at the microbiological level. Future research should focus on developing and testing such targeted strategies to mitigate infectious disease risks and enhance the quality of life for older individuals, particularly those in residential care settings.

## 1 Introduction

The composition of the microbiome changes with age (Feres et al. 2016; Odamaki et al. 2016; Asakawa et al. 2018; Lira‐Junior et al. 2018; Dashper et al. 2019; Jiang et al. 2019; Su et al. 2019; Sulyanto et al. 2019; Kazarina et al. 2023). The integrated interaction between commensal microbes and human host that forms a “holobiont” constantly varies with environmental stimuli, including diet, medication, the composition of the mucus layer, and the integrity of the epithelia. Thus, the host microbiome is dynamic (Flores et al. 2014; Aleman and Valenzano 2019). The microbial population in adulthood contributes to cellular and tissue homeostasis, while with ageing, microorganisms can also contribute to frailty and disease pathogenesis (Hergott et al. 2016; Belkaid and Harrison 2017). In the context of oral health, the oral cavity harbors one of the most diverse microbial communities in the human body, and changes in host immune responses and environmental conditions can shift oral microbial communities from symbiotic to dysbiotic states (Lamont et al. 2018).

The phylum Pseudomonadota predominates during primary dentition, whereas Bacillota becomes more predominant in the dental plaque of young adults (Xu et al. 2015). A disruption in the composition of normal oral microbiota is associated with poor oral health as well as other health problems. This has been considered under the “Ecological Plaque Hypothesis” where the oral community becomes potentially more enriched in pathogens (Kidd and Fejerskov 2013; Bertelsen et al. 2022). From a healthcare perspective, maintenance of the integrity of the microbial community benefits overall health and quality of life. In people less capable of performing or having access to oral hygiene activities, oral health declines. This has been noted in people with dementia in particular (Gao et al. 2020).

Dementia is a progressive and nonreversible neurodegenerative condition identified most commonly in people aged 65 years or older (Livingston et al. 2017). Frailty, including oral frailty, is an important outcome of a wide range of physical and mental health conditions (Newcomer et al. 2011) and is an important risk factor for dementia independent of genetic factors (Ward et al. 2022). On average people with dementia have two to eight comorbidities (Sanderson et al. 2002; Schubert et al. 2006), which may be explained either by commonality in their pathogenesis or simply a higher prevalence as measured by their frequency, for example, hypertension (Poblador‐Plou et al. 2014). Numerous studies have also highlighted compromised oral health in people with dementia (Delwel et al. 2018; Gao et al. 2020) living in residential aged‐care facilities. Thomson et al. (2018) provide specific evidence from nursing home residents in New Zealand, demonstrating that poor oral health is closely associated with cognitive decline and increased dependency. This highlights that oral health in dementia care is both a clinical concern and a key contributor to quality of life. In terms of geriatric populations, key factors associated with poor oral care include people's perception towards their dental health, poor utilization of resources, age, socioeconomic status, physical incapacity, financial capacity, avoidance (fear of needles, drilling), and inexperienced carers (Bharti et al. 2015; Khadka et al. 2021).

The caregiver's perception and knowledge towards oral care in older people are often associated with poor oral health in residential aged care. Some carers report oral care as a burden and may not follow standard protocols (Reis et al. 2011) and mention a lack of time for oral care (Stancic et al. 2016). Poor oral care, along with environmental factors, for example, diet, medication, and dry mouth, affects the distribution of oral microbial populations (Aleman and Valenzano 2019) that potentially leads to an increased presence of pathogenic microorganisms resulting in local and systemic diseases (Sedghi et al. 2021). In this paper, we hypothesized that the introduction of a structured oral care intervention could result in a change in microbiome population that may lead to improved oral health by preventing overgrowth or potential emergence of pathogenic strains. This was also done to compare typical behavior in older participants who may neglect active oral health. To study this hypothesis, two cohorts were defined (control, experimental) in which the experimental group practiced a more vigorous and evidence‐based oral hygiene program compared to a control group in which oral hygiene followed standard behavior for the residential communities.

## 2 Materials and Methods

### 2.1 Study Participants

A total of 57 participants with a clinical diagnosis of dementia were enrolled from two residential aged care centers in northern Tasmania. The mean age was 84.3 years (range: 66–95 years), and the starting proportion of male‐to‐female participants was equal in each cohort. No differences occur in age distribution. All participants were alert, understood instructions, and were not in an advanced state of dementia.

These participants were divided into control (*n* = 26) and experimental cohorts (*n* = 31). Of these participants, 27 successfully completed the 6‐week oral hygiene program and the sampling process with the control and experimental groups consisting of 10 and 17 participants, respectively. Attrition reflected the common challenges in longitudinal research with people with dementia. It was mainly due to health decline, withdrawal of consent, and changes in care.

This investigation was approved by the Human Research Ethics Committee (Tasmania) Network (permit no. H0017035). The trial inclusion criteria were the following: participants ≥ 65 years of age, male or female, and willing to consent and participate. Exclusion criteria for participants included inability to remain alert or follow simple, one‐step directions; head‐of‐bed restricted to an angle of < 30°; an inability to open their mouth; and on a prescribed nothing‐by‐mouth food and fluid intake.

### 2.2 Oral Hygiene Intervention

For the toothbrushing intervention, the “control group” maintained a normal practice in which teeth brushing was of short duration (less than 2 min) at least once per day, with less frequent cleaning of dentures (at times only once per week) for those who wear dentures. In contrast, participants in the “experimental group” participated in more active daily oral care involving toothbrushing for at least 2 min twice daily (after breakfast and dinner), along with daily denture cleaning for those who used them. This more vigorous and evidence‐based oral care period was maintained for a 6‐week duration. For both control and experimental cohorts, sampling was performed before instigation (pre‐control, preexperimental) of the active oral hygiene practices and then immediately following at the end of the experiment (post‐control, post‐experimental).

### 2.3 Oral Sample Collection

Swab samples from three oral sites, the gums, tongue, and cheeks, were collected pre‐ and post‐, for control and experimental cohorts. For this, Copan eSwab (regular flocked swab) accompanied by a collection tube containing 1 mL liquid Amies medium (480CE Copan Diagnostics) was used for the specimen collection. Briefly, the entire swab tip was gently rubbed and rotated along the surface of the sites for about 5–10 s, then placed into the collection tube, and immediately transported to the laboratory in ice boxes before storage at −80°C.

### 2.4 Extraction of DNA

Specimen collection tubes were incubated at room temperature for 15–20 min. The swabs were pressed against the wall of the holding tubes before centrifuging the liquid suspensions at 14,000 rpm for 10 min in 1.5 mL nuclease‐free microcentrifuge tubes. The supernatant (800 µL) was discarded while the remaining 200 µL volume and pellet samples were treated with 30 µL lysozyme (5%, VWR Life Science) and 100 µL of lyticase (40 U, Sigma‐Aldrich) and incubated at 37°C for 30 min with intermittent vortexing. The DNA was then extracted using the Xtreme DNA Isolation Kit (Isohelix) following the manufacturer's protocol. The extracted DNA was eluted in 100 µL elution buffer and stored at −20°C. The amount and quality of DNA was determined using Quant‐iT Qubit dsDNA HS Assay Kit (Thermo Fisher Scientific) and NanoDrop 8000 spectrophotometer (Thermo Scientific).

### 2.5 Bacterial 16S rRNA and Fungal 18S rRNA Sequencing

For analysis of the bacterial microbiome, the V3–V4 region of the 16S rRNA gene was amplified using primers 341F (5′‐CCT AYG GGR BGC ASC AG‐3′) (Lane 1991) and 806R (5′‐GGA CTA CNN GGG TAT CTA AT‐3′) (Takai and Horikoshi 2000). For mycobiome, the primers used were EUk1391F (5′ GTACACACCGCCCGTC 3′) and EukBR (TGATCCTTCTGCAGGTTCACCTAC 3′). Amplicons underwent metabarcode sequencing using the Illumina MiSeq platform. For this, the amplicons were indexed using eight PCR cycles and quantified using a KAPA library quantification kit (Roche). One hundred nanograms of DNA from each sample was used for PCR amplification, and equimolar amounts (4 nM) of each sample were pooled for sequencing. Metabarcode sequencing generated 300‐bp paired‐end reads.

### 2.6 Sequence Analyses

Pair‐end reads were joined in SEED 2 (Větrovský et al. 2018) using FastqJoin 1.1.2, and the joined files were then denoised using Mothur 1.34.4 (mean quality score ≥ 35). FASTA files of the filtered reads were generated and then processed by VSEARCH (Rognes et al. 2016) to remove putative chimeric sequences and to create a list of reads clustered at 98% similarity. The output cluster FASTA files were then combined into a single FASTA file in SEED 2, and the process of clustering and chimera removal was repeated to create a nonredundant Operational Taxonomic Unit (OTU) data set covering all the samples. The bacterial OTU list was then classified using megaBLAST v. 2.2.26+ against the Silva 138.1 nonredundant 16S rRNA gene database (Quast et al. 2013) as well as eHOMD v. 3.1 (Escapa et al. 2020) in SEED 2. The fungal sequences followed the same workflow but were classified using the UNITE v. 9.0 database (Nilsson et al. 2019).

### 2.7 Statistical Analysis

For the bacterial microbiome analysis, three samples with low reads were discarded (i.e., < 1000 reads in total each); these all came from the same participant (Table 1). Samples had 16S rRNA gene read numbers ranging from 4029 to 787326 (total read number 20480279, median 92503). For the mycobiome 18S rRNA gene sequence data set, the median read number was 2854 (total reads 1269642), and 8 low read samples (all being tongue samples) were removed, as noted in Table 1 for the downstream analyses. The data were analyzed compositionally to account for the variance in sample depth and the large number of zero values (Quinn et al. 2019). First, the Amplicon Sequence Variant (ASV) table was transformed to centered log ratios (clr) using Compositions (Palarea‐Albaladejo and Martín‐Fernández 2015) with the resemblance matrix formed by conversion of clr values to Aitchison distances. For alpha diversity calculations, the following diversity indices were obtained from the untransformed data: number of OTUs (*S*), Mergalef's richness (*d*), Pielou's evenness (*J*′), and Shannon diversity (*H*′) in Primer v. 7 (Primer‐E – Quest Research Ltd, Auckland, New Zealand). Normality was assessed using the Shapiro–Wilks test. Data factors were tested nonparametrically with the Kruskal–Wallis test. For beta diversity analysis, the resemblance matrix was initially assessed using principal coordinates analysis, multidimensional scaling (MDS), and analysis of similarity (ANOSIM). These analyses were followed up using permutational multivariate analysis of variance (PERMANOVA) and canonical analysis of principal coordinates (CAP) analysis (Anderson 2001; Anderson and Willis 2003). The number of permutations used for ANOSIM, PERMANOVA, and CAP analyses was 9999. For the PERMANOVA design, all factors tested were fixed variables and assumed an unbalanced design where fixed effects sum to zero for mixed terms. When permutation analysis was applied Monte Carlo simulation was also applied. For CAP analysis, the number of axes analyzed was constrained to *m* = ≤ 10 as advised by Ratkowsky (2016). These analyses were performed in Primer v. 7. To assess differential abundance of taxa, ANOVA‐Like Differential Expression tool (ALDEx2) (Fernandes et al. 2014) and Analysis of Compositions of Microbiomes with Bias Correction 2 (ANCOM2) (Lin and Peddada 2020) were both utilized and run in R Studio v. 2023.6. For multiple testing *p*‐values were adjusted using false discovery testing (Benjamini and Hochberg 1995) with script used from carbocation/falsediscovery (2020). The OTUs tested in this respect had to have a minimum prevalence of 10% across the whole data set (both control and experimental group data combined) and contribute overall at least 0.1% of reads. This was done to minimize spurious findings arising from patchily distributed OTUs (Nearing et al. 2022).

**Table 1:** Sampling design used to assess effect of oral hygiene interventions in a set of elderly patients.

Cohort	Sampling period	Male	Female	Cheek sample swabs	Gum sample swabs	Tongue sample swabs
Standard oral hygiene practice (control)	Pre‐intervention	3^a^	7	30 (30, 30)^b^	30 (30, 30)	30 (30, 30)
	Post‐intervention	3	7	30 (29, 30)	30 (29, 30)	30 (29, 30)
Increased oral hygiene (experimental)	Pre‐intervention	5	12	51 (51, 51)	51 (51, 51)	51 (51, 48)
	Post‐intervention	5	12	51 (51, 51)	51 (51, 51)	51 (51, 46)

^a^ The same participants were sampled before instigation of the oral hygiene practice change and immediately following the end of the experiment.

^b^ The numbers in parentheses indicate the number of samples subsequently used in the statistical analyses. Samples with a low number of reads (< 10% of the median read total) were excluded in subsequent stages of the analysis.

## 3 Results

Oral samples for both bacterial and fungal sampling were successfully obtained from 27 participants, all over 65 years of age, averaging 84.3 years of age (range: 66–95 years). This amounted to an overall 47% success rate from the original starting number of participants. The control group consisted of 10 (3 male and 7 female) participants, and the experimental group included 17 (5 male and 12 female) participants. The age distribution was similar across both groups, with no statistically significant differences in mean age (Table 1).

### 3.1 Bacteriome and Mycobiome Population in the Oral Cavity of People With Dementia Living in Residential Care Facilities

**Figure 1:**
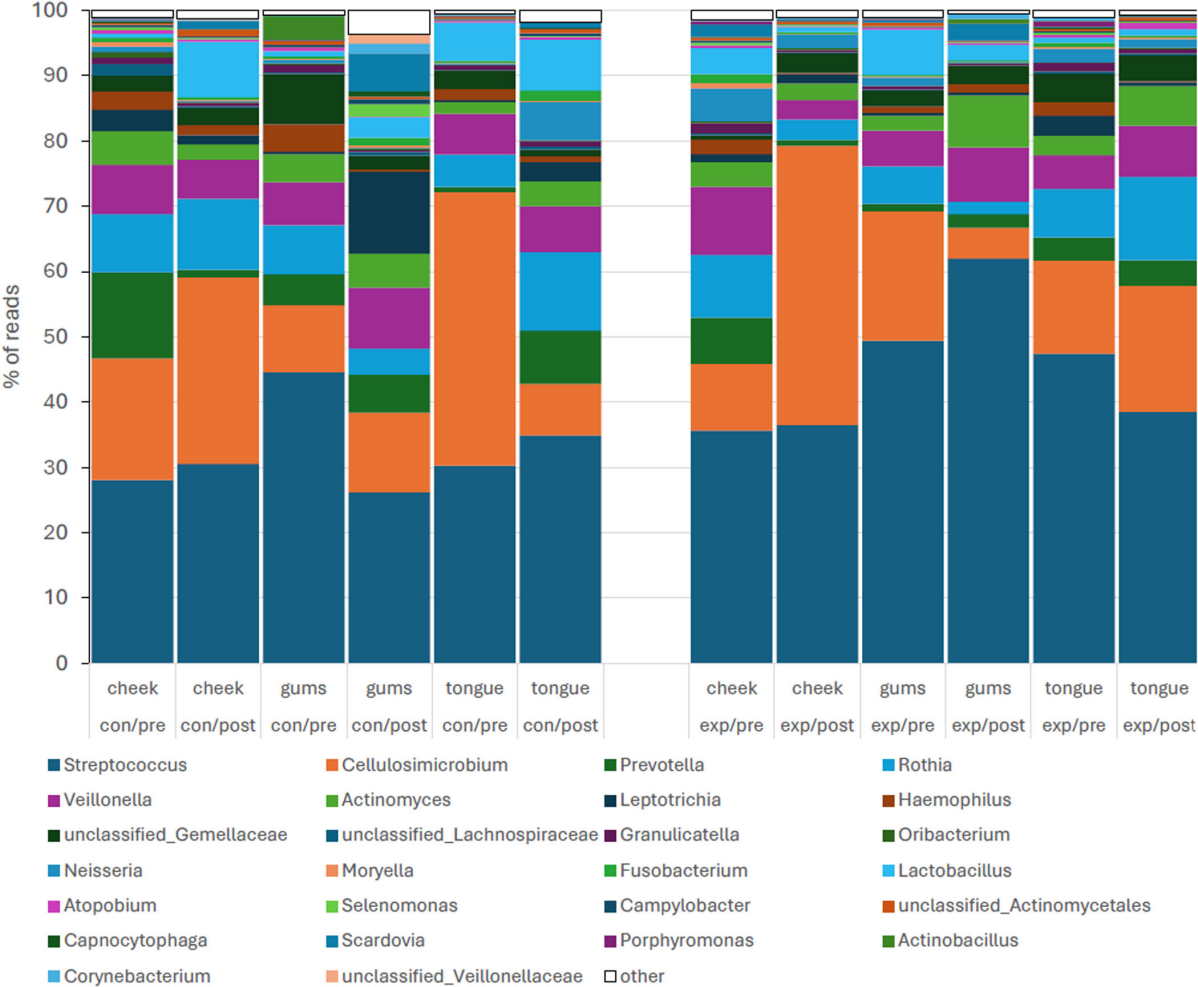
Taxonomic composition of the oral cavity bacterial community of older participants in an oral hygiene program (EXP, experimental group) and those practicing standard oral care (CON, control group). Samples were collected before (PRE) and after (POST) the 6‐week program period. The structure for oral sites from which samples were swabbed is shown for the given treatment groups.

**Figure 2:**
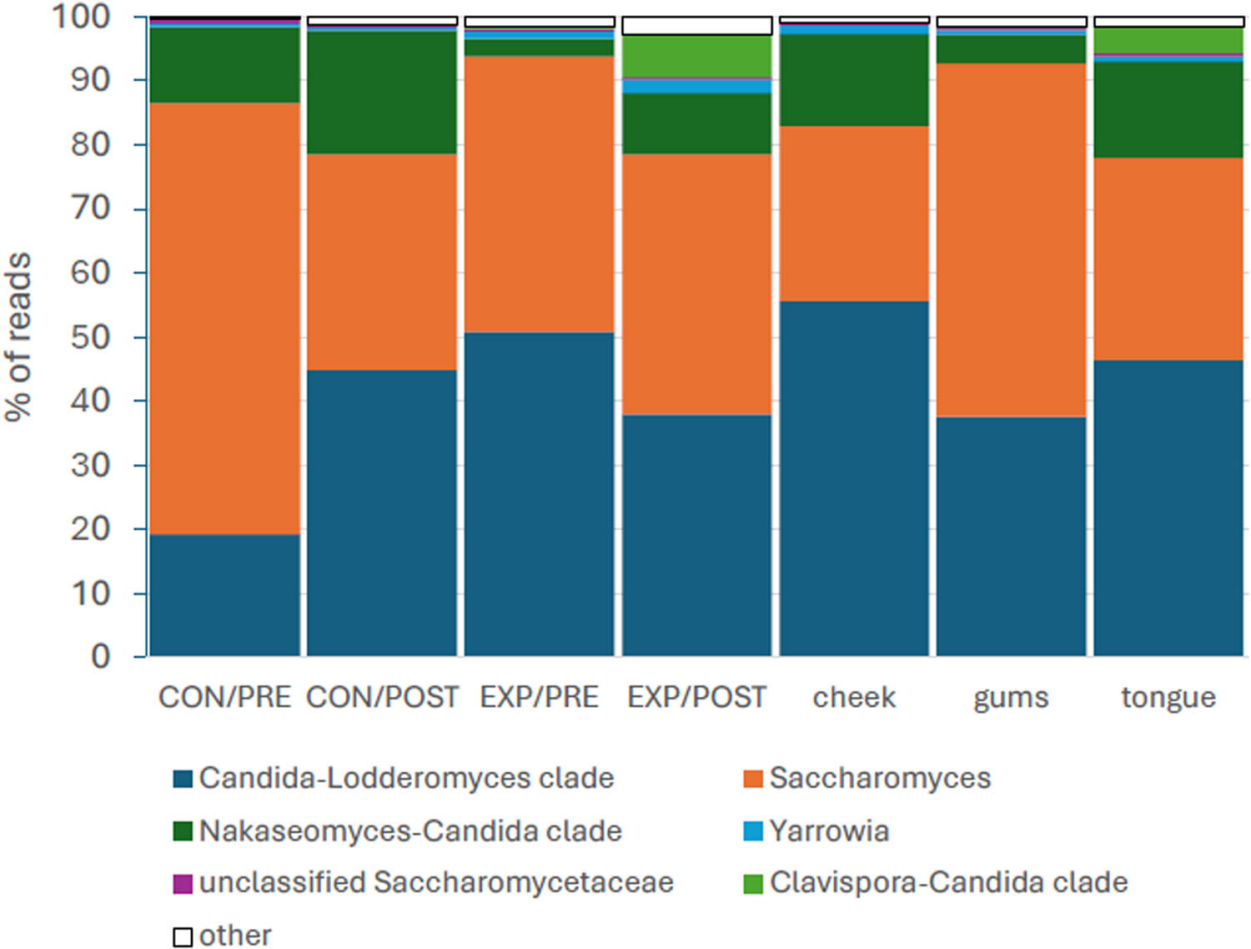
Taxonomic composition of the oral cavity fungal community of older participants in an oral hygiene program (EXP, experimental group) and those practicing standard oral care (CON, control group). Samples were collected before (PRE) and after (POST) the 6‐week program period. Due to a lack of alpha diversity and compositional differences, the CON and PRE tongue, gums, and cheek samples were pooled combined and then compared to respective PRE and POST treatment groups.

The predominant bacteria sampled (Figure 1) included mainly Bacillota, Actinomycetota, Bacteroidota, Fusobacteriota, and Pseudomonadota. The average contribution of the main genera between the control and experimental cohorts were *Streptococcus* (32.4%, 44.9% of reads), *Veillonella* (7.1%, 6.7%), *Actinomyces* (3.8%, 4.3%), *Gemella*/unclassified Gemellaceae (3.1%, 2.9%), *Granulicatella* (0.7%, 0.8%), *Lactobacillus* (and related genera) (4.5%, 2.6%), *Cellulosimicrobium* (19.9%, 18.5%), *Rothia* (8.1%, 6.8%), *Scardovia* (1.3%, 0.9%), *Prevotella* (5.6%, 3.1%), *Leptotrichia* (3.5%, 1.2%), *Fusobacterium* (0.7%, 0.5%), *Haemophilus* (1.9%, 1.1%), and *Neisseria* (1.3%, 2.0%). Oral mycobiome largely consisted of yeast taxa of class Saccharomycetes (Figure 2). Saccharomycetes outnumbered the read abundances of other types of fungi by two orders of magnitude. For the control and experimental groups, respectively, the main taxa included *Saccharomyces* (43.2%, 47.6%), the *Candida‐Lodderomyces* clade (both 39.8%), the *Nakaseomyces‐Candida* clade (14.9%, 5.7%), the *Clavispora‐Candida* clade (0.03%, 2.0%), and *Yarrowia* (0.5%, 2.0%). The most abundant non‐Saccharomycetes taxa were *Cystofilobasidium*, *Malassezia*, *Aspergillus*, and *Alternaria*, which contribute less than 0.25% of reads each.

### 3.2 Impact of Increased Oral Hygiene on Bacterial Richness and Diversity in the Oral Cavity of Older Participants in Residential Care

**Figure 3:**
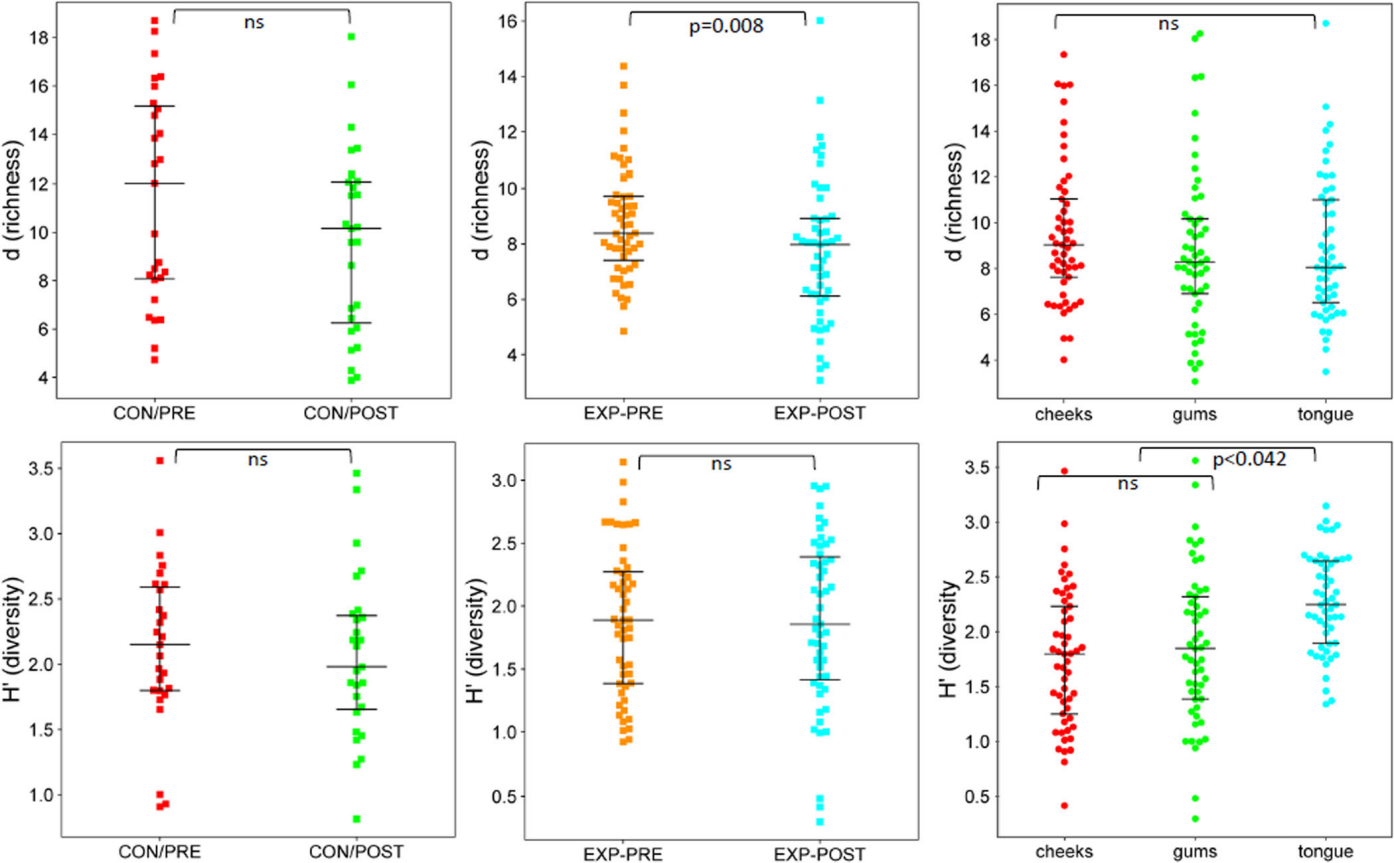
Alpha diversity swarm plots for the oral cavity bacterial community of older participants in an evidence‐based oral hygiene program (EXP, experimental group) and those practicing standard oral care (CON, control group). Samples were collected before (PRE) and after (POST) the 6‐week program period. The significance of the species richness, represented as Mergalef's richness (*d*), and taxonomic diversity, represented as Shannon's Index (*H*′), is shown (ns = not significant, *p* > 0.05).

**Figure 4:**
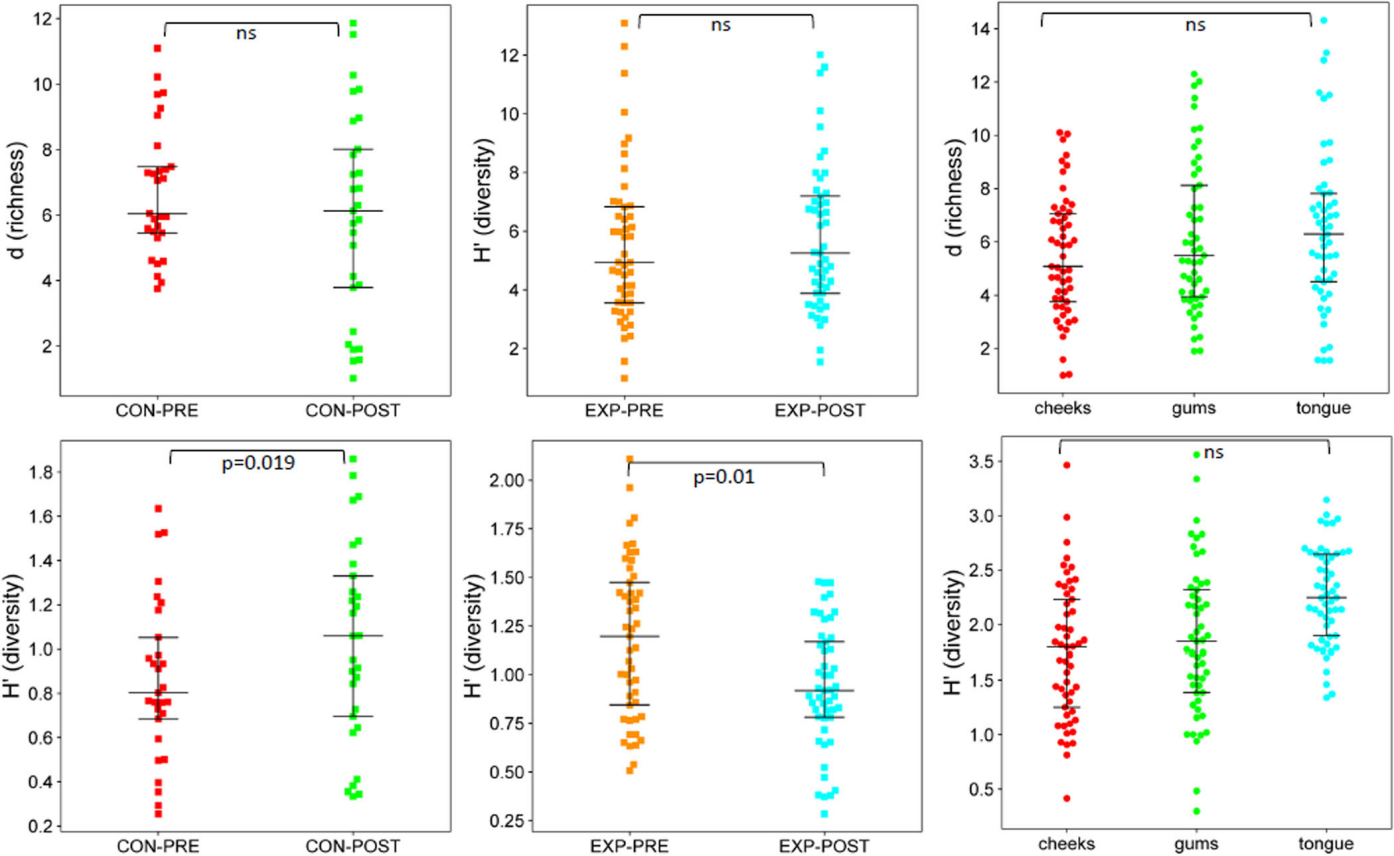
Alpha diversity swarm plots for the oral cavity fungal community of older participants in an oral hygiene program (EXP, experimental group) and those practicing standard oral care (CON, control group). Samples were collected before (PRE) and after (POST) the 6‐week program period. The significance of the species richness, represented as Mergalef's richness (*d*), and taxonomic diversity, represented as Shannon's Index (*H*′) is shown (ns = not significant, *p* > 0.05).

Diversity comparisons shown include Mergalef's richness (*d*) and Shannon diversity values for bacterial (Figure 3) and fungal oral communities (Figure 4) for the control and experimental groups comparing pre‐ and post‐intervention sampling periods and oral sampling sites (cheeks, gums, and tongue). The other diversity measures used (*S*, *J*′) correlated closely with d and *H*′ data and are thus not shown.

Examining the effect of alpha diversity in the bacterial communities sampled involved looking at both experimental effects on two independent cohorts and the more specific effect of selective oral site sampling. Looking at the data as a whole, the sampling process before (pre‐intervention) and after (post‐intervention) the experimental period, combining both control and enhanced oral hygiene cohorts had no effect on taxonomic diversity distribution (*J*′, 0.42 ± 0.12, 0.43 ± 0.14; *H*′, 1.94 ± 0.66, 1.99 ± 0.61, p‐adj = 1.0). However, the species richness was reduced slightly (~17% on average) in the post‐intervention groups compared to the pre‐intervention groups (*S*, 113 ± 42, 94 ± 0.39, p‐adj = 0.011; *d*, 9.7 ± 3.2, 8.4 ± 3.2, p‐adj = 0.032). In that respect, the effect of the changes in oral hygiene practice can only be assessed by a distinct and independent response in the control and experimental groups. For the control group, this would be expected to involve no change in alpha diversity parameters since oral hygiene practiced follows the status quo. Though a 20% reduction in richness was observed at the end of the experiment for the control group, there was high variability across this data set (*S*, 132 ± 58 to 106 ± 43; *d*, 11.3 ± 4.2, 9.5 ± 3.6, *p* = 0.077). The taxonomic distribution was unaffected (*J*′, both sets 0.45 ± 0.11, *H*′, 2.13 to 2.17 ± 0.61, *p* > 0.35). For the experimental group, the starting and ending diversity was lower than the control group's, thus directly comparing between the groups suggested there were no gross changes in diversity (Figure 3). The richness did decrease by approximately 15% at the end of the experiment (*S*, 101 ± 25, 86 ± 35, *p* = 0.008; *d*, 8.8 ± 2.0, 7.6 ± 2.6, *p* = 0.026); however, the taxonomic distribution was unaffected (*p* > 0.42). Oral sites sampled contributed to the overall variances in taxonomic distributions in the bacterial microbiome data set; however, richness between cheeks, gums, and tongue was not significantly different for all treatment groups (Figure 3, *p* > 0.07). The tongue samples had the highest taxonomic diversity while cheek samples had the least (cheek vs. tongue: *J*′, 0.38 ± 0.13 vs. 0.50 ± 0.09; *H*′, 1.75 ± 0.62 vs. 2.27 ± 0.41, adj *p* < 0.0001). Within the experimental group, this trend was more obvious (Figure 3). Gender also had minimal to no effect on diversity or richness measures, especially not aided by the number of female participants overshadowing that of males (Table 1).

### 3.3 Effect of Increased Oral Hygiene Practice on Oral Fungal Diversity in Older Participants in Residential Care

For the mycobiome data set, the number of reads obtained for the same samples was 16‐fold less than for the bacterial microbiome which may reflect lower biomass levels, especially since the 18S rRNA gene operon repeats in fungi can be as high as 150–200 copies (Pendrak and Roberts 2011; Sharma et al. 2022) compared to equivalent copy numbers in oral bacteria (1–10 copies), (Su et al. 2019). Alpha diversity measurements showed no differences between pre‐ and post‐intervention sample sets (*p* > 0.72), oral sites (*p* > 0.84), and gender (> 0.093). Control sample data fungal richness and diversity were, on average, greater than that of the experimental data sets (Figure 4), though overall, this was not found to be large enough to be significant (*p* > 0.1). For the control pre‐ and post‐intervention sample sets, richness was not altered (*S*, 61 ± 7 vs. 53 ± 6; *d*, 6.7 ± 2.0 vs. 6.2 ± 3.2, *p* > 0.28). Between control group pre‐ and post‐intervention sample groups, taxonomic diversity values increased slightly, though the change was only statistically marginal (*J*′, 0.22 ± 0.10 to 0.32 ± 0.18, *p* = 0.019, *H*′, 0.86 ± 0.36 to 1.08 ± 0.49, *p* = 0.08). For the experimental cohort oral hygiene intervention also had no impact on fungal richness (Figure 4, *p* > 0.43); however, taxonomic diversity was reduced slightly (*J*′, 0.34 ± 0.15 to 0.27 ± 0.15, *p* = 0.01; *H*′, 1.17 ± 0.41 to 0.94 ± 0.32, *p* = 0.006) though the endpoint diversity values were in the same range as that found for the control samples.

### 3.4 Community Compositional Changes Arising From Increased Oral Hygiene Practice

**Figure 5:**
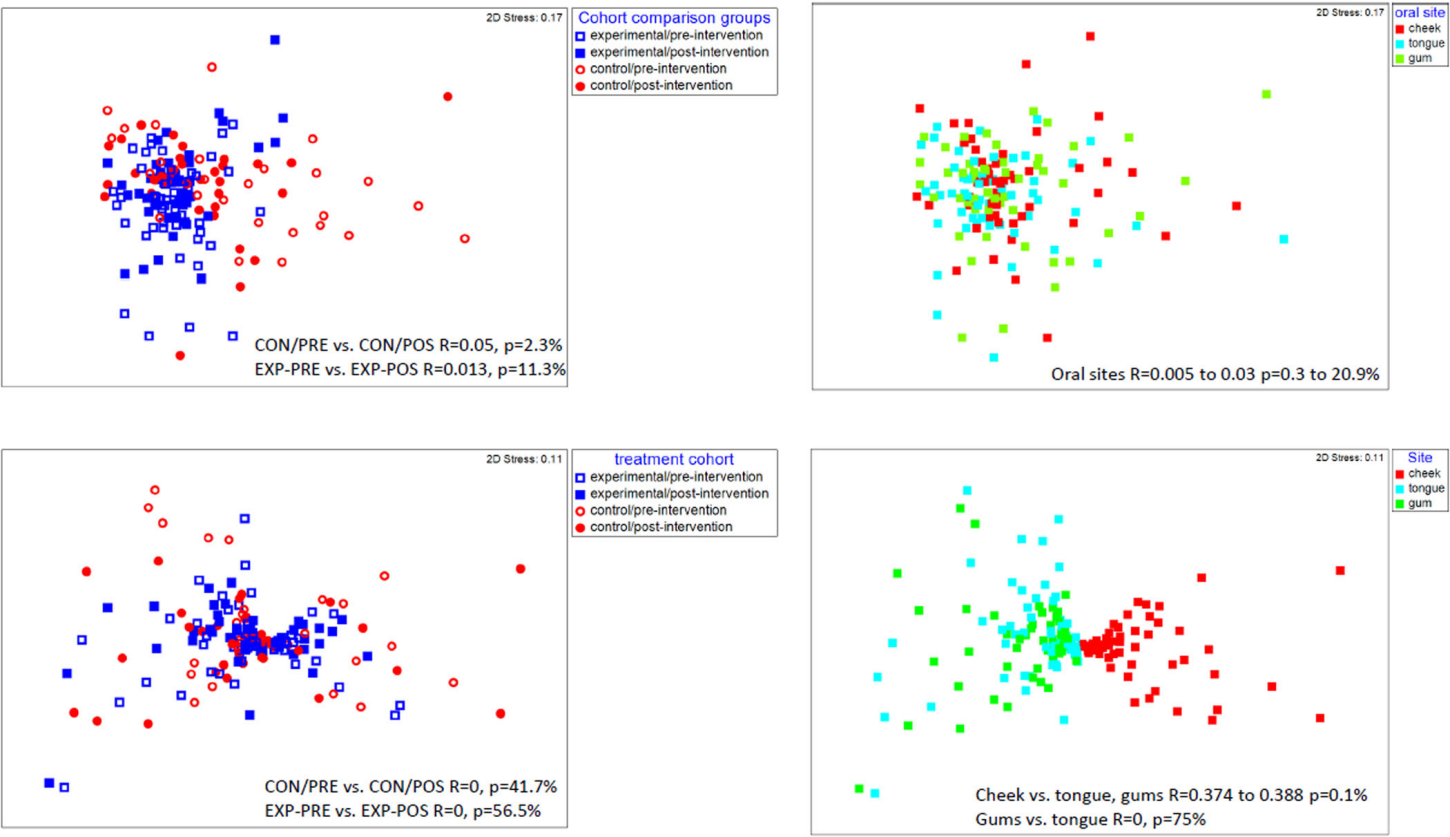
MDS plot of oral cavity bacterial (top two plots) and fungal (bottom two plots) representing communities of older participants in an oral hygiene program (EXP, experimental group) and those practicing standard oral care (CON, control group). Samples were collected before (PRE) and after (POST) the 6‐week program period. The right‐hand plots depict treatment group comparison, whereas the left‐hand plots depict oral site comparisons. The *R*‐value denotes the results from ANOSIM and includes the % significant values (*n* = 9999 permutations). 2D stress values indicate the goodness‐of‐fit in the MDS ordination.

MDS and ANOSIM analysis were used to establish a priori differences based on the metadata used to structure the compositional data, transformed and compared as Aitchison distances. For the bacterial data, as suggested by the lack of taxonomic diversity differences, the communities highly overlapped (Figure 5). Post‐ and pre‐intervention, oral sites, and gender all demonstrated this trend (*R* = 0.005–0.091) while complete separateness occurs when *R* = 1. Only marginal significances at most (significance, 0.2%–20.9%) were observed for these comparisons. When control and experimental samples were compared, the least overlap was observed (*R* = 0.165, *p* = 0.1%), which suggests the sample community structures differ to an extent between the two participant groups, though the OTU compositions were similar (see below). However, the oral hygiene practice intervention did not produce any impact on community structural differences (Figure 5, *R* = 0.013–0.05, *p* = 2.3%–11.3%). PERMANOVA and residual analysis were also used to test the data, mostly to determine what degree do the factors compared contribute to the variances in the data set. From the analysis, the cohorts (control vs. experimental) and the oral sites had only made minor contributions, covering 2% and 5% of the data, respectively. Pair‐wise analysis indicated the pre‐ and post‐intervention comparisons for the control groups were not significant (*p* = 0.072) as would be expected. For the experimental groups, PERMANOVA pair‐wise analysis suggests only a marginal difference (*p* = 0.023), which in light of the MDS and ANOSIM data, does not demonstrate any differences of note given the low input to the variance. CAP analysis supports this, indicating that correlation‐based classifying of the cohort/intervention sample groups was only, on average, 52% successful, essentially indicating any differences were random effects. For confidence that the microbiome factor groups are nonrandom, where communities are distinct statistically, the classification ideally should be a high percentile value supported by multivariate‐capable significance testing.

For the mycobiome data, when treatment groups are compared, there is an obvious high overlap (Figure 5) in MDS plots. ANOSIM revealed there was no significant separation between intervention groups, gender, control versus experimental cohorts, or from the effect of oral hygiene intervention in the experimental group (*R* = 0–0.062, *p* = 3%–59.9%). Cheek fungal profiles overall were distinct from gum and tongue profiles (Figure 5, *R* = 0.42–0.45, *p* = 0.1%). There was no difference between gums and tongue samples (*R* = 0, *p* = 75%). The MDS and ANOSIM data, followed up by PERMANOVA and CAP analysis, categorically showed there was no impact on the mycobiome deriving from the oral hygiene program (*p* = 0.57, 42% successful classification of groups). Oral sites contributed 23% of the variance compared to the treatment cohort, contributing less than 1%. Also, 94% of cheek samples could be successfully classified against gums and tongue samples, which were much less distinguishable (30%–45% misclassification). Based on PERMANOVA data, the variation in the microbiome and mycobiome data sets was mostly derived from participant samples. For the microbiome, this was estimated from partitions of the variance at 49.3% (pseudo‐*F* = 8.4, *p* < 0.0001), including interactions with site and treatment group. As much as 78% of the variance is explained (Table S1). It should be noted that the participants were different in the control and experimental groups, so variance estimates for ID versus treatment are approximated. In the case of the mycobiome data, this was also the case; however, interactions occurring between participants and the oral sampling sites are much more influential in structuring the data (Table S2). Collectively, participant and other factor interaction was estimated at making up 70% of the total variances.

### 3.5 Structure of the Oral Microbiome Between Control and Experimental Cohorts

Comparing the control and experimental groups (combining all oral sites) only *Streptococcus* and *Campylobacter* (0.40%, 0.12%, respectively) abundances differed significantly (ALDeX2 and ANCOM2, p‐adj < 0.05, Cohen's *d* = 0.54–0.57) and even then, the fold‐difference is feeble when considered on a logarithmic scale (1.4–threefold, 0.1–0.5 log units).

## 4 Discussion

Teeth brushing and denture cleaning serve a similar function to skin washing and cleansing by removing excess microbial biofilm accumulation, including cellular biomass and microbial end‐products that have accumulated on the surfaces that are being physically cleaned (Edmonds‐Wilson et al. 2015; Skowron et al. 2021). Although their effect on soft tissues (gums, tongue, and cheek) is more indirect, regular cleaning helps reduce the load and transfer of potentially pathogenic microorganisms, thereby lowering the risk of oral and systemic infections. Although the use of cleaning implements may, in some instances, facilitate pathogen transfer, the benefits of mechanical biofilm removal outweigh the risks (Carling et al. 2023).

Poor oral hygiene is known to increase bacterial diversity and facilitate colonization by periodontal (Bertelsen et al. 2022) and respiratory pathogens (Asakawa et al. 2018). This study implemented a 6‐week oral hygiene protocol in two separate cohorts (control and experimental) involving more teeth brushing and denture cleaning compared to the status quo. Conclusively, we could not detect any substantive effects on species (OTU) richness, while trends for taxonomic diversity were different between control and experimental cohorts, albeit marginal in nature, and likely reflecting the cohort differences and thus not relevant to the oral hygiene intervention. Our data suggest the practice does not alter the suite of bacterial taxa present particularly in the absence of additional interventions such as antimicrobial toothpastes (Paqué et al. 2021), oral rinses (Radzki et al. 2022), or probiotics (Homayouni Rad et al. 2023).

In individuals who may be experiencing dementia and are dependent on others for their oral care, toothbrushing has been associated with both beneficial and adverse outcomes such as increased gum bleeding, periodontitis, tooth loss, stomatitis, and candidiasis, and proposed as an infection source for related diseases (Gao et al. 2020; Jung and Jang 2022). Toothbrush bacterial assemblages have been found to include a mix of environmental and oral bacterial taxa some of which could have pathogenic potential or act as reservoirs of antimicrobial resistance genes (e.g., *Acinetobacter*, *Stenotrophomonas*, and *Candida albicans*) (Blaustein et al. 2021; Shang et al. 2020); however, the actual risk of pathogen transfer remains unclear.

An unexpected finding in this study was the high abundance of *Cellulosimicrobium*, a genus typically reported in salivary microbiomes in healthy patients at much lower levels (0.1%–0.3%) (Wang et al. 2016). Its predominance here may reflect the mucosal swabbing technique used, which captured microbes from saliva and soft tissues rather than tooth surfaces. It was also a feature of the participant groups since it did not appear in the laboratory sequence control samples. Other taxa such as *Streptococcus* were consistent with oral community norms (Kazarina et al. 2023).

The oral hygiene intervention in the experimental group did not affect the overall abundances of the more predominant genera (0.1% or greater read contribution, > 10% prevalence). Changes in less abundant taxa were considered spurious since the prevalence across participants was always patchy arising from participant variations. Differential abundance analysis is limited under such conditions, and results may vary significantly depending on the statistical model used (Nearing et al. 2022). Some abundant taxa, for example, the genera *Leptotrichia*, *Neisseria*, *Scardovia*, and *Actinobacillus* were highly variable between participants, and this tendency is greatly inflated for low‐abundance taxa.

Fungal taxa differences were more evident, the clearest being the abundances of the *Clavispora‐Candida* clade and *Yarrowia*. Most of these differences are attributable to oral site variations and also subject to considerable variation in each of the treatment groups. Tongue samples were quite variable in terms of read distributions, possibly related to generally lower sequence yields, compared to cheek and gum samples. Many fungal taxa also tend to vary across the data set due to participant oral community structure uniqueness. Comparing the post‐intervention and pre‐intervention samples for the experimental group using differential abundance analysis, there was no convincing evidence that particular fungal taxa were impacted by improved oral hygiene.

The null hypothesis is obtained by the observation that the slight alpha diversity changes in the bacterial microbiome of the control and experimental groups followed similar trends, where as distinct changes were expected in the experiment group. While richness was a more variant feature of the bacterial microbiome, the overall taxonomic distribution remained relatively stable within each cohort, except when specific oral sample sites were compared in isolation. The same result was found for the mycobiome, though oral sites demonstrated differences in fungal communities that tended to overshadow the impact of the intervention, as suggested by the PERMANOVA data. It should be noted that the difference between cheek, gums, and tongue samples varied by cohort and did not represent systematic differences. This may be happenstance when analyzing samples from small cohorts. Overall, the general lack of differences could partly be due to abundant and prevalent bacterial and fungal OTUs within the communities being resilient to physical and chemical perturbances.

Measuring the effect of an oral hygiene practice on microbiomes is challenging due to several reasons. First, individual people have dynamic and different oral community structures (Flores et al. 2014; Nearing et al. 2020) that make it hard to overcome the variations inherent in a small cohort study. Second, the vigor of application of practice change likely varies between individuals and over the narrow time frame of experiments. Behavioral differences thus may not offset natural variations in oral microbiomes arising from diet, lifestyle, aging, and other potential variations that are more influential on short temporal scales (Willis et al. 2022; Kazarina et al. 2023). Data from this study revealed that only relatively small proportions of the variance could be detected (via PERMANOVA) related to the control and experimental cohort research design. This is perhaps unsurprising given that extensive metadata analysis of the microbiomes of a large saliva data set only yielded small individual contributions to the variation (Nearing et al. 2020). The majority of the variation instead lay with individuals, oral sites, and interactions with other unknown factors. As stated by Nearing et al. (2020), new biological markers or metadata need to be found to be able to overcome the high level of variations contributed by individuals. Targeted and practical oral hygiene interventions for those with special needs may need to be formulated or if available (e.g., Strickland et al. 2023) appropriately validated. Finally, the main oral bacteria found in the study were likely highly adherent and prevalent on oral cavity mucosal surfaces and occurred in high numbers in saliva. Also, in terms of growth rates and biomass, the bacteria were typically highly fit for the oral cavity environment, that is, adapted to the prevailing temperature, pH, fluid ionic composition, oxygen fluxes, and nutrients. This ecological relation means that oral taxa have a high degree of resilience to disturbance. The review by Fine and Schreiner (2023) describes this resilience to disturbance in terms of landscape ecology. Thus, the inherent tenacity and high growth rates of oral bacteria make it challenging to significantly perturb or remove oral bacterial and fungal populations (Wade 2021) especially with only short‐term interventions. Impacts on microbiome community structure by extrinsic factors that are intermittent and not continual would lead to only short‐term and small proportional changes in major taxa. Total removal or exclusion of taxa due to the expansion of aggressive and harmless colonizers to the host would require more targeted and intense forms of disruption. In this study low‐abundance taxa tended to be much more variable and patchier in distribution between individuals and sampling sites, and their contributions were not testable. Though low‐abundance microbiome taxa can be influential in some instances in terms of human biology (Pust and Tümmler 2022), the reason they are not examined is to enable effective analysis of what is rather noisy data (Cao et al. 2021).

Several limitations affected this study. First, no clinical oral health data (e.g., plaque indices, periodontal scores) were collected. This was an ethical decision to minimize participant distress in a population of older adults with moderate dementia. As a result, the microbiome data may reflect only mucosal and salivary colonizers.

Second, the cohort size was modest with fewer than 40 individuals per group, detecting statistically significant shifts in microbial abundance at the OTU level requires a large effect size (Cohen's *d* > 0.8). This is dependent on the species/OTU being present in most individuals as well as being relatively abundant. We accounted for this by setting constraints on the analysis (OTUs with 10% minimum presence amongst samples and an abundance minimum of 0.1% of total reads). Our analysis found no OTUs with a Cohen's *d* above 0.6 (maximum observed *d* = 0.57), indicating that systematic alterations in the microbiome do not occur. This lack of significance may reflect both biological variability and insufficient statistical power, particularly given the complexity and individuality of oral microbiomes in older adults with dementia.

Third, the sequencing method (16S rRNA V3–V4) limited taxonomic resolution, making it difficult to assess species‐ or strain‐level changes, especially for low‐prevalence or pathogenic taxa.

However, the inherent variability between individuals combined with the minimal effect of the intervention suggests that larger cohorts and deeper sequencing (i.e., greater read depth) would be needed to detect more subtle microbial shifts. Larger‐scale studies, using a simple sampling strategy, that is, saliva from control and experimental cohorts, can yield differences at the genus level such as that by Malan‐Müller et al. (2024). This study identified genus‐level differences in a community cohort of over 300 individuals, which underscores the value of sample size in detecting oral microbiome patterns in relation to mental and systemic health conditions.

Finally, in our study, a correlation between microbial composition and “pathogenic potential” could not be established based on the data obtained. We argue that such a correlation is inherently challenging and would require assumptions that go beyond the scope of this small‐scale study. A more precise identification of community members would be necessary, which could be achieved through long‐read sequencing technologies such as Oxford Nanopore. In addition, establishing virulence potential would require either inference from curated databases such as the Virulence Factor Database (VFDB) or experimental validation. The latter might include advanced culturomics approaches as described by Khelaifia et al. (2023), or comprehensive metagenomic analysis. However, both approaches are resource‐intensive and not feasible within the current study's design and scale.

## 5 Conclusions

While the intervention did not produce significant shifts in oral bacterial or fungal community structures of older adults in residential care, the outcome highlights the inherent resilience of the oral microbiome and the limitations of short‐term, nontargeted interventions. Other limitations included the modest cohort size and substantial interindividual variability, limited sequencing resolution, and short intervention duration, which limited the statistical power to detect smaller or site‐specific microbial changes.

Furthermore, the primer set used (V3–V4) provided insufficient resolution to identify microbes at the species level, constraining interpretation of possible pathogen dynamics. A future study would benefit from utilizing long‐read sequencing to yield both greater resolution and more accurate species identification (Esberg et al. 2024).

It is also important to recognize the complexity of oral health management in dementia care. Oral hygiene should not be viewed in isolation; changing brushing practices alone is unlikely to overcome the multifaceted drivers of poor oral health outcomes in this population. Nonetheless, the study contributes valuable baseline data and underscores the need for more intensive, personalized, and possibly longer term strategies to modulate oral microbiota in vulnerable aged populations. This requires improved oral hygiene approaches that can move more substantially away from current approaches.

## Author Contributions

S.K., S.G., L.C., A.K., L.R.G., and S.S.B. performed the experiments. S.G. and J.P.B. analyzed the data. S.S.B., S.G., S.K., L.R.G., and J.P.B. wrote the manuscript. J.P.B. and S.G. share joint second authorship.

## Ethics Statement

The Human Research Ethics Committee (Tasmania) Network (permit no. H0017035).

## Conflicts of Interest

The authors declare no conflicts of interest.

## Supporting information


**Table S1:** PERMANOVA output for mycobiome community structure examining the influence of oral site (cheeks, gums, tongue), treatment (control/pre‐intervention, control/post‐intervention, experimental – pre‐intervention, experimental – post‐intervention), and participant ID. The analysis does not separate control and experimental groups thus at the lowest levels samples are not fully replicated for the calculation of ID × treatment. **Table S2:** PERMANOVA output for mycobiome community structure examining the influence of oral site (cheeks, gums, tongue), treatment (control/pre‐intervention, control/post‐intervention, experimental – pre‐intervention, experimental – post‐intervention), and participant ID. The analysis does not separate control and experimental groups thus at the lowest levels samples are not fully replicated for the calculation of ID × treatment.

## Data Availability

The data that support the findings of this study are available on request from the corresponding author. The data are not publicly available due to privacy or ethical restrictions. Sequence data were deposited under BioProject PRJNA1073289 in the NCBI Sequence Read Archive.
