# Supplementary material for: The Effect of 6‐Week Advanced Oral Care on Oral Microbiome and Mycobiome Composition in People With Dementia Living in Residential Aged Care
Source: Clin Exp Dent Res. 2025 Sep 8;11(5):e70212. doi: 10.1002/cre2.70212 (PMC12415712; doi:10.1002/cre2.70212)
Supplement: Supplementary file 1 — Table S1: PERMANOVA output for mycobiome community structure examining the influence of oral site (cheeks, gums, tongue), treatment (control/pre‐intervention, control/post‐intervention, experimental – pre‐intervention, experimental – post‐intervention), and participant ID. The analysis does not separate control and experimental groups thus at the lowest levels samples are not fully replicated for the calculation of ID × treatment. Table S2: PERMANOVA output for mycobiome community structure examining the influence of oral site (cheeks, gums, tongue), treatment (control/pre‐intervention, control/post‐intervention, experimental – pre‐intervention, experimental – post‐intervention), and participant ID. The analysis does not separate control and experimental groups thus at the lowest levels samples are not fully replicated for the calculation of ID × treatment. [file CRE2-11-e70212-s001.docx]

**Table S1** PERMANOVA output for mycobiome community structure examining the influence of oral site (cheeks, gums, tongue), treatment (control/pre-intervention, control/post-intervention, experimental – pre-intervention, experimental – post-intervention), and participant ID. The analysis does not separate control and experimental groups thus at the lowest levels samples are not fully replicated for the calculation of ID × treatment.

| Source | est. % of partitioned variance | df | SS | MS | Pseudo-F | P(perm) | permutations | P(MC) |
| --- | --- | --- | --- | --- | --- | --- | --- | --- |
| Oral site | 1.4 | 2 | 1441.6 | 720.78 | 3.1643 | 0.0001 | 9862 | 0.0001 |
| Treatment | 2.3 | 3 | 2341.6 | 780.52 | 3.4266 | 0.0001 | 9819 | 0.0001 |
| Participant ID | 49.3 | 26 | 49724 | 1912.4 | 8.396 | 0.0001 | 9602 | 0.0001 |
| Site × treatment | 1.7 | 6 | 1672.2 | 278.69 | 1.2235 | 0.0756 | 9787 | 0.0709 |
| Site × ID | 13.4 | 52 | 13498 | 259.58 | 1.1396 | 0.0219 | 9608 | 0.0231 |
| ID × treatment | 15.5 | 26 | 15661 | 602.36 | 2.6445 | 0.0001 | 9631 | 0.0001 |
| Residuals | 10.4 | 46 | 10478 | 227.78 |  |  |  |  |
| unexplained | 6.0 |  | 6103.6 |  |  |  |  |  |
| Total | 100 | 161 | 100920 |  |  |  |  |  |

**Table S2** PERMANOVA output for mycobiome community structure examining the influence of oral site (cheeks, gums, tongue), treatment (control/pre-intervention, control/post-intervention, experimental – pre-intervention, experimental – post-intervention), and participant ID. The analysis does not separate control and experimental groups thus at the lowest levels samples are not fully replicated for the calculation of ID × treatment.

| Source | est. % of partitioned variance | df | SS | MS | Pseudo-F | P(perm) | permutations | P(MC) |
| --- | --- | --- | --- | --- | --- | --- | --- | --- |
| Oral site | 9.1 | 2 | 2378.5 | 1189.2 | 17.113 | 0.0001 | 9897 | 0.0001 |
| Treatment | 1.9 | 3 | 488.09 | 162.7 | 2.3412 | 0.0019 | 9902 | 0.0022 |
| Participant ID | 24.8 | 26 | 6459.4 | 248.44 | 3.575 | 0.0001 | 9746 | 0.0001 |
| Site × treatment | 2.4 | 6 | 619.91 | 103.32 | 1.4867 | 0.0423 | 9846 | 0.0318 |
| Site × ID | 24.4 | 52 | 6342.5 | 121.97 | 1.7552 | 0.0001 | 9765 | 0.0001 |
| ID × treatment | 10.0 | 26 | 2592.1 | 99.694 | 1.4346 | 0.0025 | 9795 | 0.0033 |
| Residuals | 12.3 | 46 | 3196.7 | 69.494 |  |  |  |  |
| unexplained | 15.1 |  | 3928.8 |  |  |  |  |  |
| Total | 100 | 161 | 26006 |  |  |  |  |  |
